# Loke zupa decoction attenuates bronchial EMT-mediated airway remodelling in chronic asthma through the PI3K-Akt/HIF-1α signaling pathway

**DOI:** 10.1080/13880209.2023.2244543

**Published:** 2023-09-01

**Authors:** Jiani Liu, Li Li, Xue Han, Yue Chen, Juanjuan Diao

**Affiliations:** a College of Pharmacy, Xinjiang Medical University, Urumqi, P.R. China; b Xinjiang Medical University, Urumqi, P.R. China

**Keywords:** BEAS-2B cells, cytokine, lung, E-Cadherin, N-Cadherin

## Abstract

**Context:**

Loke zupa decoction (Lok) is a well-established classic Chinese folk remedy for asthma.

**Objective:**

We sought to investigate the effect and mechanism of Lok on asthma airway remodelling and provide novel insights for the prevention and treatment of asthma.

**Materials and methods:**

For *in vitro* experiments, BEAS-2B cells were assigned into six groups: Control, TGF-β1 (10 μM), TGF-β1 + Lok-20, TGF-β1 + Lok-40, TGF-β1 + Lok-80 μg/mL and TGF-β1 + SB431542 (5 μM). CCK8 and wound healing assays were performed. For *in vivo* experiments, 60 female BALB/c mice were randomly divided into 5 groups: Control, model, Lok-4.55, Lok-9.1, and DEX group. Lok was administrated by gavage during the challenge stage for 8 consecutive weeks (4.55 and 9.1 g/kg/day). We investigated airway inflammation and airway remodelling in the lungs and verified the activation status of EMT-related markers and the PI3K-Akt/HIF-1α signalling pathway.

**Results:**

*In vitro*, Lok efficiently inhibited TGF-β1-induced BEAS-2B cell proliferation ability (cell viability 165% vs. 105%) and migration (migration areas 85% vs. 35%) without affecting their normal growth (IC50 274.2 µg/mL at 48 h). *In vivo*, Lok effectively protected mice from asthma, as evidenced by decreased histological damage and level of cytokines in BALF (IL-4, IL-13 and TGF-β1) by 17%–77%. Mechanistic research revealed that Lok reduced the levels of EMT-related molecules and significantly downregulated the PI3K-Akt/HIF-1α signalling pathway.

**Discussion and conclusions:**

Our findings provide novel insights into the protective effect of Lok on asthma and the underlying mechanisms, providing a theoretical basis and potential treatment possibilities for this patient population.

## Introduction

Airway remodelling is characterized by structural changes in the airway, including the thickening of airway walls, subepithelial collagen deposition and excessive mucus secretion (Bullone and Lavoie 2020). It has been established that respiratory symptoms in asthma patients are largely caused by airway obstruction. Accordingly, it is crucial to find effective approaches that can reverse the ongoing remodelling process (Grainge et al. 2011; Gosens and Grainge 2015). Repeated inflammation of airway epithelial cells can lead to EMT development and differentiation into myofibroblasts, which exacerbates subepithelial fibrosis and contribute to airway remodelling in asthma. Many proteins reportedly play an important role in EMT, including N-cadherin and E-cadherin, representing epithelial and mesothelial features. Inflammatory and structural cells of the airway release cytokines and growth factors that promote airway inflammation, which promotes remodelling. Transforming growth factor-β1 (TGF-β1) reportedly plays an important role in allergic inflammation and airway remodelling, produced by various types of cells in the lung, such as airway epithelial cells and other lung pro-inflammatory cell infiltrates (Yao et al. 2019). Studies have shown that the PI3K/Akt pathway is broadly involved in various cellular processes, such as cell differentiation and proliferation, inflammation, metabolism and apoptosis (Chung 2017). Accumulating evidence suggests the key role of the PI3K/Akt pathway in the immune response and remodelling in the airways, contributing to the pathological changes of asthma (Wei et al. 2022). In this respect, it has been reported that inhibiting the PI3K/Akt pathway by Akt dephosphorylation could reduce the expression of hypoxia-inducing factor HIF-α and vascular endothelial growth factor VEGF, thereby reducing the permeability of respiratory mucosa, inhibiting respiratory oedema and contraction, and reducing airway remodelling in asthma (Dahlin et al. 2018). To our knowledge, airway remodelling in asthma has been largely understudied, warranting further studies on the mechanism of EMT in airway remodelling for the prevention and treatment of asthma.

Currently, the treatment of asthma relies on drugs such as inhaled glucocorticoids (ICS) and β2 agonists. However, the effectiveness of conventional therapies remains limited due to the complex aetiology of asthma. In addition, many drugs are associated with serious side effects, including an increased risk of cardiovascular disease, gastrointestinal damage and metabolic disorders (Derendorf et al. 2006; Caramori et al. 2022). Chinese medicine, which emphasizes a holistic approach to treatment, offers alternative possibilities. Lok is a traditional folk medicine widely used in northwest China, consisting of *Hyssopus officinalis* L. (Lamiaceae). and roots of *Iris halophila* Pall. (Iridaceae) (Abduwaki et al. 2017). Rosmarinus acid has been identified by liquid chromatography–tandem mass spectrometry (LC–MS/MS) as the main active ingredient of *H. officinalis*, while luteolin, iris and apigenin are the main active ingredients of the roots of *I. halophila* (Wuniqiemu et al. 2021; Aihaiti et al. 2022). Preliminary studies have shown that Lok could regulate the TH1/TH2 cytokine balance to reduce airway inflammation in mouse models of asthma (Mohammadtursun et al. 2020; Wei et al. 2016). Besides, it has been reported that the ethanolic extract of Iris halophila Pall, the main drug in Lok, prevents bronchial asthma by inhibiting mitogen-activated protein kinase/NF-κB inflammatory signalling (Yuan et al. 2019), and the chemical components of Lok have strong anti-inflammatory and antioxidant effects (Li et al. 2021). The above studies suggest that Lok can effectively treat asthma. Nonetheless, no studies have hitherto reported on the effect of Lok on asthma airway remodelling. Therefore, we conducted *in vivo* and *in vitro* experiments to evaluate its effect on airway remodelling.

## Materials and methods

### Preparation of Lok

The preparation of Lok was conducted based on a previous study (Xie et al. 2022). In this study, the whole herb of *H. officinalis* and the roots of *I. halophila* were pulverized and mixed with water decoction. The mixture was then subjected to water decoction. The resulting decoction was filtered, concentrated, and dried to obtain a dry extract. The yield of the extraction process was determined to be 17.52%. The identification of the two herbs used in Lok was carried out by Dr. Perrida Ablizi, School of Pharmacy, Xinjiang Medical University. A reference specimen was preserved in the School of Pharmacology, Xinjiang Medical University (accession numbers xwyz20190612 and xwyz20190610).

### Cell culture, treatment

Human bronchial epithelial cells (BEAS-2B) were purchased from Fenghu Biotechnology (Wuhan, China) and cultured in DMEM medium (Gibco, UK) with 10% FBS (Gibco, UK) at 37 °C in a humidified incubator containing 5% CO_2_. To induce EMT, the BEAS-2B cells were treated with 2.5, 5 and 10 ng/mL TGF-β1 (Sigma-Aldrich, USA) for 24, 48 and 72 h.

### Cell viability and proliferation assays

The viability of BEAS-2B cells incubated for 48 h at different concentrations, including 0, 2.5, 5, 10, 20, 40, 80, 160 and 320 μg/mL, was assessed using a Cell Counting Kit-8 (Bioss, China). A total of 5000 cells in 100 μL of complete medium (DMEM + 10% FBS) were added to the wells of a 96-well plate. The CCK-8 reagent (10 μL) was diluted with DMEM at a 1: 10, and 100 μL of the working solution was added to the wells. Absorbance values were measured at 450 nm after 2 h of incubation. For the proliferation assay, three replicates were treated with the above treatments.

### Wound healing assay

A wound healing assay was used to determine whether Lok inhibited cell migration. BEAS-2B cells were seeded at a density of 5 × 10^4^ cells/well in 6-well plates overnight. Cell monolayers were scratched with a 100 μL plastic needle and washed thrice with PBS. Cells were then treated with 10% FBS medium supplemented with TGF-β1 (10 ng/mL) in the presence or absence of Lok (20, 40, 80 μg/mL) or SB431542 (HY-10431, MCE, USA) for 48 h. The morphological changes of cells were observed by microscopy (OLYMPUS, IX73P1F, Japan).

### Ovalbumin (OVA)-induced asthma model

Sixty female BALB/c specific-free pathogen mice (18–20 g, 5–6 weeks old) were purchased from the Experimental Animal Center of Xinjiang Medical University. All experimental protocols involving animals were approved by the Ethics committee of Xinjiang Medical University (Approval ID: IACUC-20210805-08). Mice were acclimatized and fed in a standard facility for 1 week with free access to food and water. They were randomly divided into five groups (*n* = 12). (1) Control group, challenged with phosphate-buffered saline (PBS) for 8 weeks; (2) Mod group, challenged with OVA for 8 weeks; (3) High-dose Lok decoction group (9.1 g/kg); (4) Lok decoction low-dose group (4.55 g/kg); (5) Dexamethasone (Dex) group (0.5 mg/kg), referred to as Lok-4.55, Lok-9.1, and Dex in this study. The doses of Lok decoction were equivalent to 4.55 and 9.1 g of herb/kg body weight in raw, in accordance with previous studies (Xie et al. 2022). The asthma group was sensitized with 100 µg OVA (Sigma Aldrich, USA) and 1 mg aluminium hydroxide (Sigma Aldrich) in 0.2 mL PBS on days 0, 7 and 14, respectively. From day 21, PBS was inhaled by nebulization with 5% OVA for 30 min/d every other day for 8 consecutive weeks. From day 21, OVA-sensitized mice were given 0.2 mL Dexamethasone (0.5 mg/kg) or Lok decoction (4.55 g/kg, 9.1 g/kg) by gavage 1 h before 5% OVA challenge, while the control group was given normal saline.

### Collection of bronchoalveolar lavage fluid (BALF) and cell counts

Twenty-four hours after the last challenge, each mouse was weighed and injected with 2% pentobarbital sodium (Sigma-Aldrich, St. Louis, MO, USA) at a dose of 50 mg/kg; to collect BALF, 1.6 mL of PBS was injected into the lungs in two equal portions and repeated three times by gentle aspiration, with a fluid recovery of 80% considered satisfactory. The cell suspension was centrifuged (1500 rpm, 4 °C, 10 min), and leukocytes in BALF were counted using a whole blood analyzer (BC-5000Vet, Mindray Co.

### Quantification of cytokines and growth factors

BALF was used undiluted. For the assessment of protein content of IL-4 (E-EL-M0043c), IL-13 (E-EL-M0727c) and TGF-β1 (JL12223-96T), ELISA was performed using the corresponding kits from Elabscience (Wuhan, China) and Jianglabic (Shanghai, China).

### Determination of HPY

The right lung tissue of mice was obtained by alkaline hydrolysis (Nanjing Jiancheng, China) to determine the content of HPY. The tissue was put into a test tube, and the hydrolysate was added. The mixture was then hydrolyzed in a boiling water bath. After hydrolysis, an indicator was added, and the pH value was adjusted accordingly. The mixture was diluted with double-steamed water and thoroughly mixed. 3 mL of hydrolysate was added to the activated carbon and centrifuged at 3500 rpm for 10 min. The supernatant was added to the corresponding reagent and mixed according to the kit instructions. After centrifugation in a water bath, the supernatant was analyzed at 550 nm for absorbance value detection.

### Morphological changes in the airways

The left lungs of mice were fixed in 4% paraformaldehyde, embedded, and cut into 3 μm thick sections. Hematoxylin–eosin (H&E) staining was used to assess the extent of airway inflammation and airway remodelling. Masson trichrome staining was used to detect subepithelial collagen deposition. PAS-stained sections were used to observe airway mucus secretion.

### Western blot analysis

Total protein was extracted from mouse lung tissue and BEAS-2B cells, quantified, and diluted to the same concentration with 4× loading buffer. Proteins were separated on 10% or 8% SDS-PAGE gels and transferred to PVDF membranes. (Millipore, Billerica, MA, USA). After blocking with TBS-Tween 20 containing 5% skim milk for 3 h at room temperature, the membranes were incubated with primary antibodies for E-cadherin (Cat. No. 20874-1-AP), N-cadherin (Cat. No. 22018-1-AP), α-SMA (Cat. No. 14395-1-AP), AKT (Cat. No. 60203-2-lg), phospho-AKT (Cat. No. 66444-1-lg), HIF-1α (Cat. No. 20960-1-AP) and GAPDH (Cat. No. 10494-1-AP) were obtained from Proteintech (Wuhan, China), Anti-PI3K (bs-10657R) was ordered from Bioss (Beijing, China). Anti-p-PI3K (AP0854) was obtained from ABclonal (Wuhan, China) overnight at 4 °C with GAPDH as an internal control. The membrane was then washed with 1× TBST, incubated with the corresponding secondary antibody for 1 h, and washed again with 1× TBST. The density of the protein bands was analyzed using ImageJ software.

### Quantitative real-time PCR analysis

Total RNA from mice lung tissue was extracted with TRIzol reagent (Takara, Shiga, Japan), and then the cDNA was reverse transcribed. qRT-PCR was performed to measure mRNA levels relative to GAPDH expression. The RT-qPCR conditions were as follows: 95 °C 3 min, 95 °C 10 sec, and 65 °C 30 sec for 45 cycles. The expression of target messenger RNA (mRNA) was quantified by the 2^−ΔΔCt^ method and GAPDH expression was used for normalization. The required primer sequences are shown in Table 1 (Sangon Biotech, Shanghai, China).

**Table 1.** Sequence of primers.

Primers	Forward	Reverse
N-Cadherin	CAATGGAGACTGCACGGACG	TCCCGCCGTTTCATCCATAC
E-Cadherin	ACCGGAAGTGACTCGAAATGATGT	CTTCAGAACCACTGCCCTCGTAAT
α-SMA	CCCAGATTATGTTTGAGACC	TCCAGAGTCCAGCACAATAC
GAPDH	GGTTGTCTCCTGCGACTTCA	GGGTGGTCCAGGGTTTCTTA

### Statistical analysis

All experimental data were expressed as the mean ± standard deviation, and statistical analysis was performed using SPSS 24.0 (SPSS Inc., Chicago, IL, USA). One-way analysis of variance (One-way ANOVA) was used to compare multiple groups. LSD was used for the pairwise comparison of variance, and Dunnett’s T3 method was used for the pairwise comparison of uneven variance. A *p*-value of <0.05 indicated statistical significance.

## Results

### Effect of Lok on viability of BEAS-2B cells

**Figure 1.**
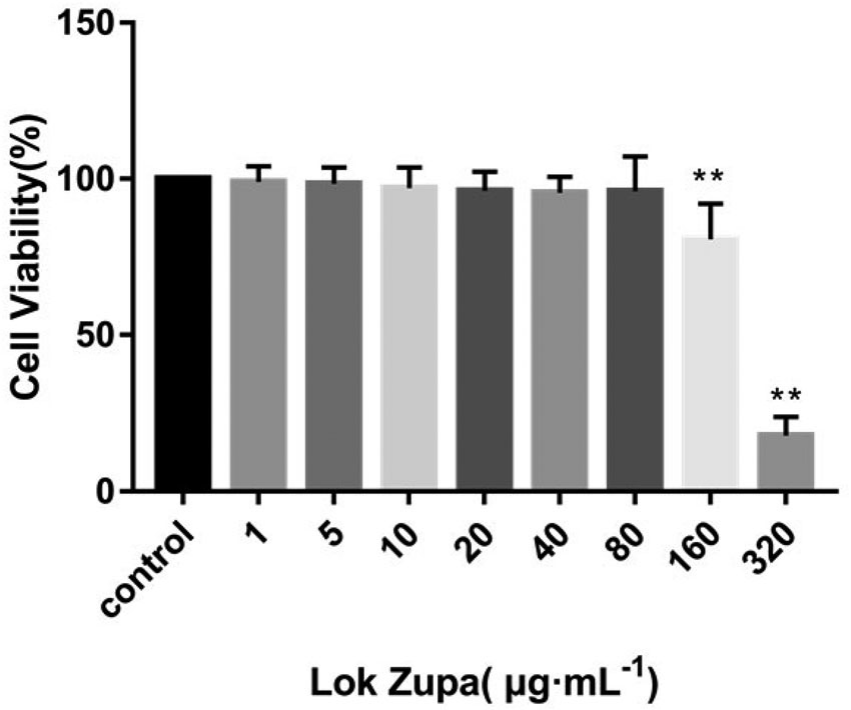
Effect of Lok on viability of BEAS-2B cells. BEAS-2B cells were seeded in 96-well plates and stimulated with Lok at concentrations of 0, 1, 5, 10, 20, 40, 80, 160 and 320 µg/ml for 48 h. The cell viability was measured by CCK-8 assay. The obtained data were expressed as mean ± SD. n = 3, *p < 0.05, **p < 0.01, vs. con.

Initially, we sought to determine suitable concentrations of Lok on BEAS-2B, and the viability of cells was examined by the CCK-8 kit assay. Results showed that when the concentration of Lok was lower than 160 µg/mL, the survival rates at 48 h were greater than 80.7 ± 11.2% (Figure 1). The IC_50_ value was 274.2 µg/mL. As such, it was determined that the study used 20, 40 and 80 µg/mL as the experimental concentrations.

### Optimal conditions for TGF-β1 induction of EMT in BEAS-2B cells

**Figure 2.**
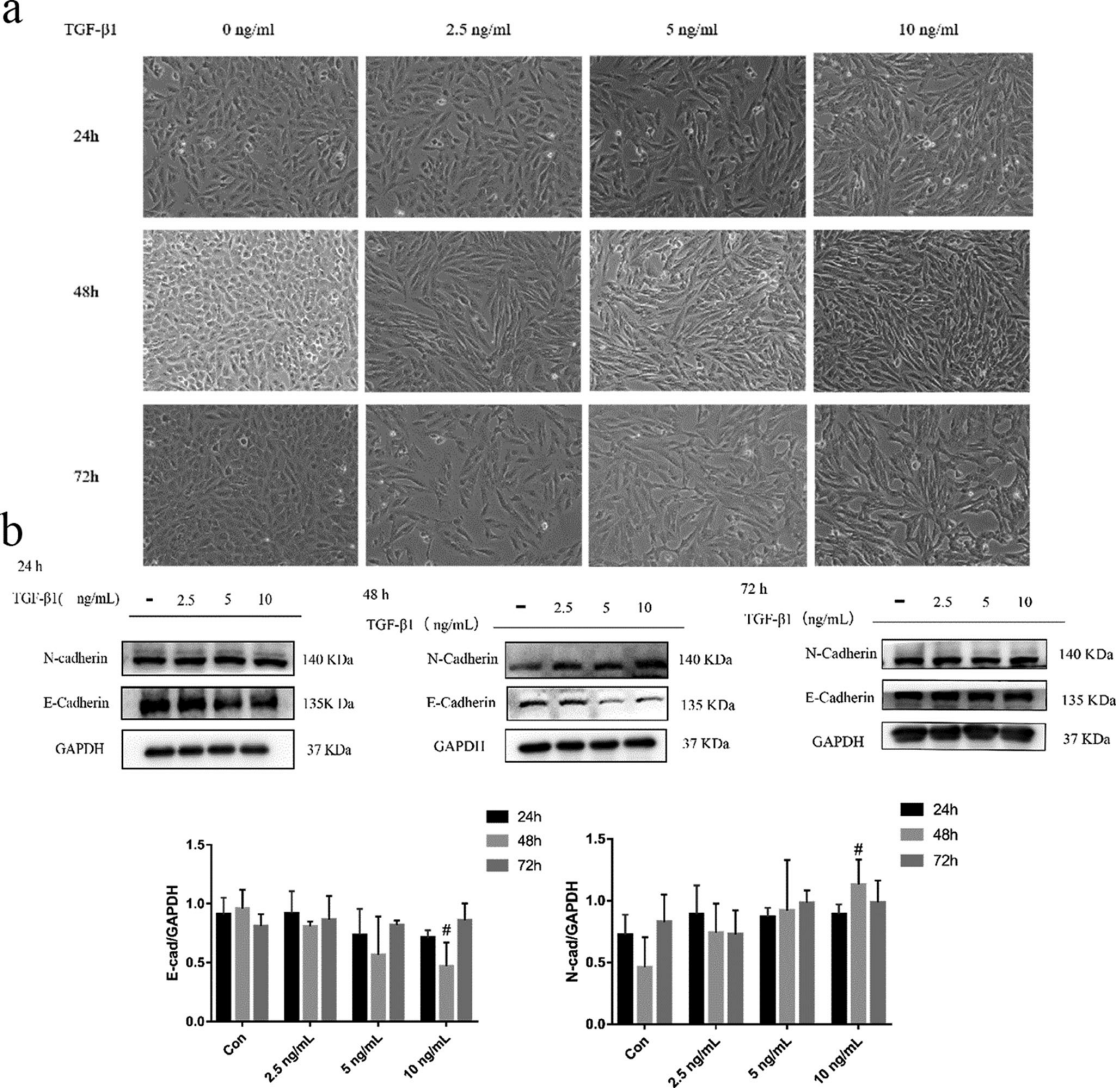
TGF-β1-induced EMT in BEAS-2B cells. (a) Morphological changes in BEAS-2B cells induced by different concentrations and duration of action of TGF-β1 (magnification × 200); (b) Effects of different concentrations and duration of action of TGF-β1 on EMT-related proteins in BEAS-2B cells. Immunoblotting (upper panel) and quantitative analysis (lower panel) of E-cadherin and N-cadherin in BEAS-2B cells treated with different concentrations of TGF-β1 for different intervals. GAPDH was used as an internal standard. The obtained data were expressed as mean ± SD. n = 3, *p < 0.05, **p < 0.01, vs. con.

To study the effect of TGF-β1 on BEAS-2B cells, the cells were treated with 2.5, 5 and 10 ng/mL of TGF-β1 for 24 h, 48 h and 72 h. The morphological changes in each group were observed under a microscope. As shown in Figure 2(a), the control cells exhibited round or oval shapes with close contact between cells. With an increased TGF-β1 dose, the cells exhibited a spindle shape and proliferated. EMT markers were detected by Western blot. The epithelial marker E-cadherin decreased most significantly in cells when the TGF-β1 concentration was 10 ng/mL for 48 h, while the mesenchymal marker N-cadherin was upregulated (Figure 2(b)). Overall, treatment of BEAS-2B cells with 10 ng/mL TGF-β1 for 48 h was the optimal concentration and duration of action.

### Lok inhibits TGF-β1-induced cell proliferation and migration of BEAS-2B

**Figure 3.**
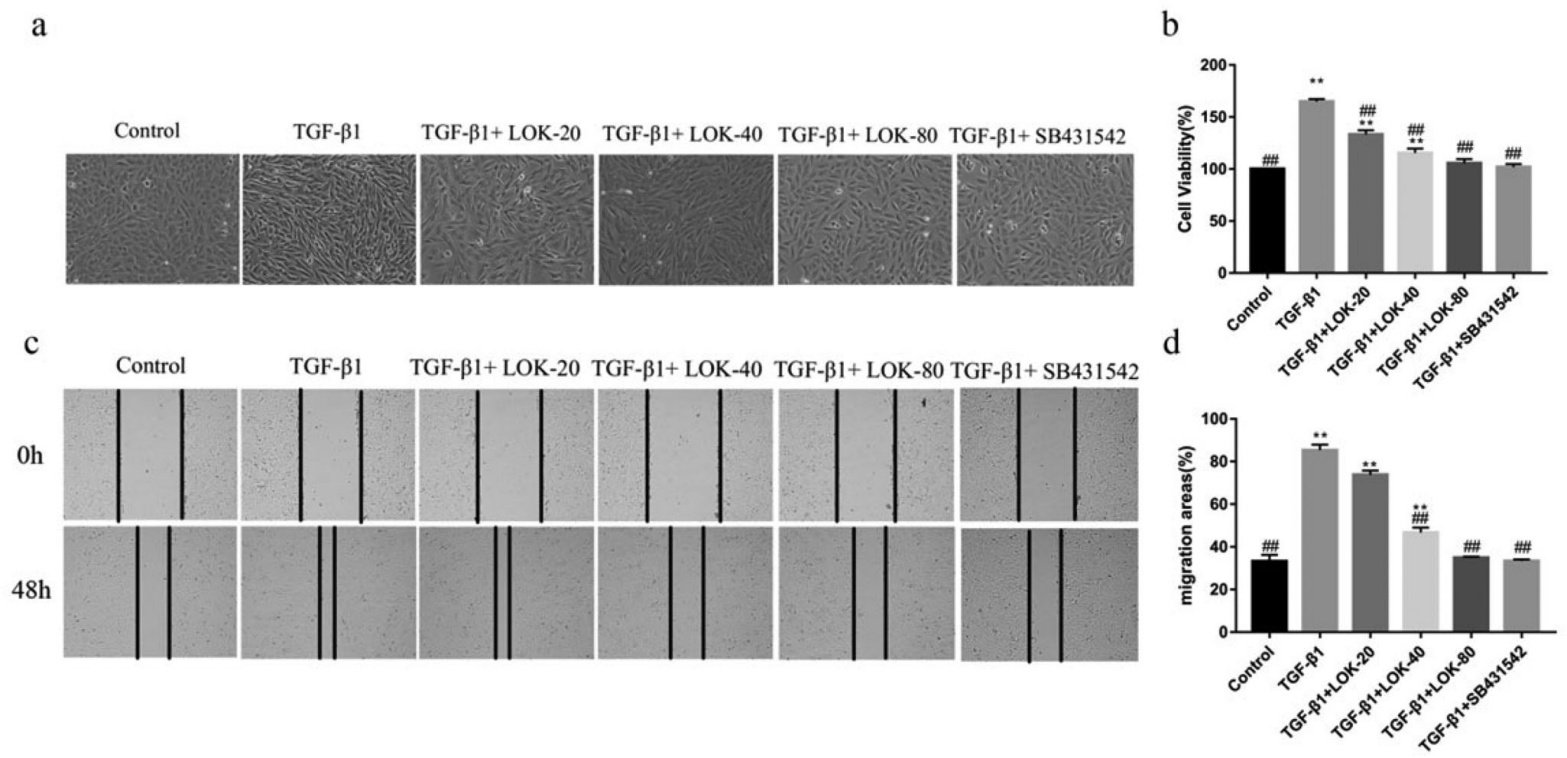
Lok inhibited cell migration and proliferation in TGF-β1-treated BEAS-2B cells. (a) Phase-contrast microscopy showed that cells stimulated with TGF-β1 exhibited a spindle-shaped fibroblast-like morphology with reduced cell-to-cell contact, while Lok and SB431542 improved cell morphology. (b) Cell proliferation capacity was analyzed by CCK-8 assay. (c) After the cells reached 90% confluence, they were scraped horizontally with a sterile 100 μl pipette tip. Images of the wound area were captured at 0 and 48 hours post-stimulation. (d) The migration area was measured to analyze cell migration. One-way ANOVA followed by Dunnett's multiple comparisons test was used. *p < 0.05, **p < 0.01, vs. control group, ^#^*p* < 0.05, ^##^*p* < 0.01, vs. TGF-β1 group. The data are presented as mean ± SD, n = 3.

The migration and proliferation ability was increased in TGF-β1-induced EMT cells to explore the effect of Lok on the proliferation and migration ability of TGF-β1-induced cells. The CCK-8 assay further confirmed the inhibitory effect of Lok (20, 40, 80 µg/mL) or SB431542 (5 µM) on TGF-β1-induced proliferation. As shown in Figure 3(a), TGF-β1 (10 ng/mL) increased BEAS-2B cell proliferation after treatment for 48 h, but Lok (20, 40, 80 µg/mL) or SB431542 (5 µM) significantly suppressed the proliferation ability. Phase-contrast microscopy showed that cells stimulated with TGF-β1 exhibited spindle-shaped fibroblast-like morphology with reduced cell-to-cell contact, and these changes could be improved with Lok treatment (Figure 3(d)). The wound healing assay results showed that TGF-β1 increased cell migration ability after treatment for 48 h in BEAS-2B cells, while Lok (20, 40, 80 µg/mL) or SB431542 (5 µM) suppressed the cell migration ability (Figure 3 (c and d)). Taken together, Lok suppressed TGF-β1-induced migration and proliferation ability in BEAS-2B cells.

### Lok regulates TGF-β1-induced expression of EMT markers

**Figure 4.**
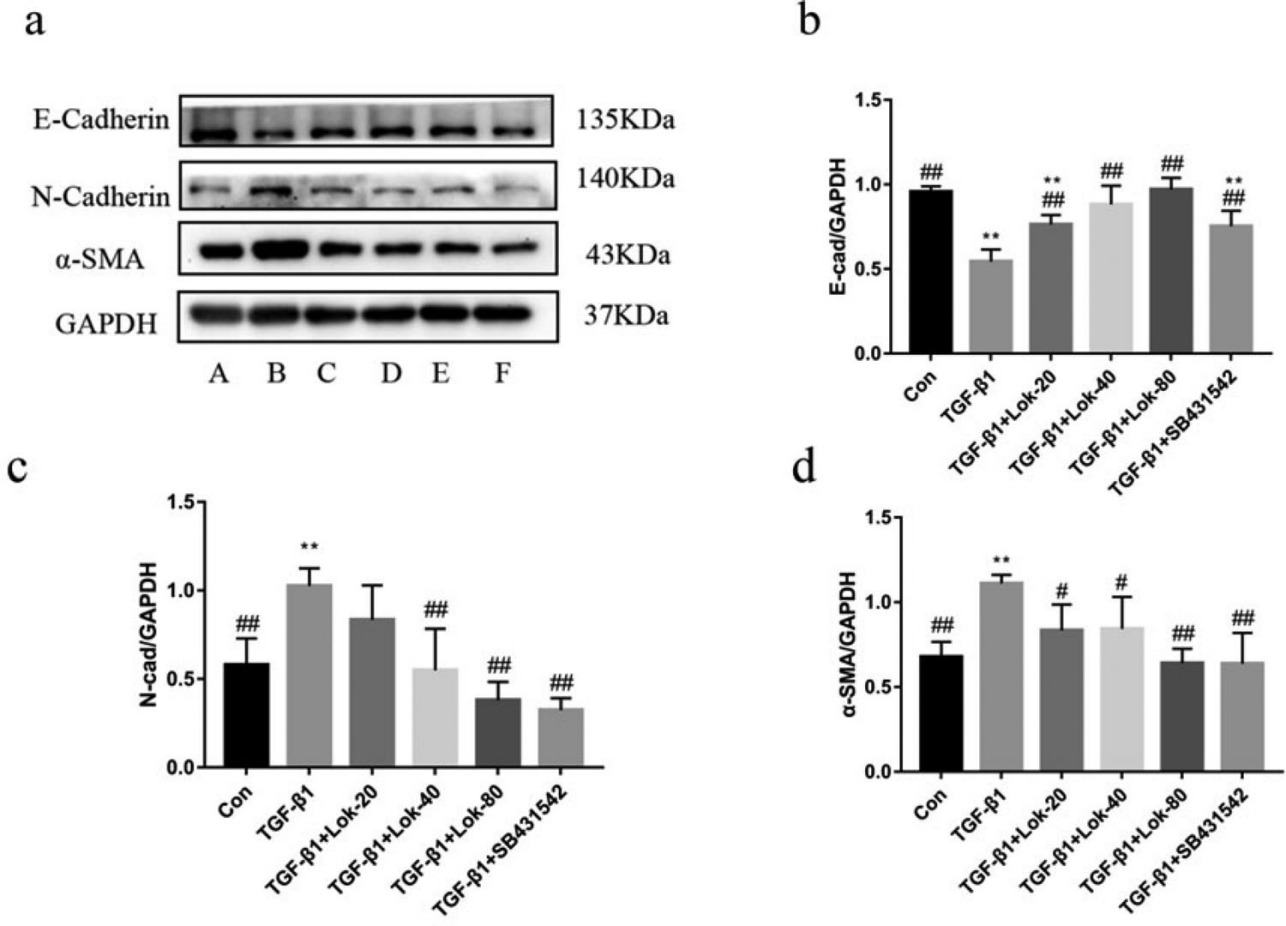
Lok inhibits EMT in BESA-2B cells exposed to TGF-β1. (a) The expression of E-cadherin, N-cadherin and α-SMA was detected by Western blot. A: control, B: TGF-β1 (10 ng/mL), C: TGF-β1 with Lok (10 ng/mL and 20 µg/mL respectively), D: TGF-β1 with Lok (10 ng/mL and 40 µg/mL, respectively), E: TGF-β1 with Lok (10 ng/mL and 80 µg/mL, respectively), F: TGF-β1 with SB431542 (10 ng/mL and 5 μM, respectively). Ratios of E-cadherin (b), N-cadherin (c) and a-SMA (d) intensity. The obtained data are expressed as mean ± SD. n = 3, *p < 0.05, **p < 0.01, vs. con, ^#^*p* < 0.05, ^##^*p* < 0.01, vs. TGF-β1.

It is well-established that E-cadherin, α-SMA and N-cadherin are important proteins in the EMT process. Importantly, we found that TGF-β1 could decrease E-cadherin expression and increase N-cadherin and α-SMA levels. The Western blot assay assessed whether Lok could inhibit EMT-related protein expression. Results showed that TGF-β1 (10 ng/mL) upregulated the expression of N-cadherin and α-SMA and downregulated E-cadherin after treatment for 48 h. However, these changes could be reversed after treatment with Lok (20, 40, 80 µg/mL) or SB431542 (5 µM) (Figure 4).

### Lok reversed TGF‑β1‑induced EMT in BEAS-2B cells through the PI3K-Akt/HIF-1α pathway

**Figure 5.**
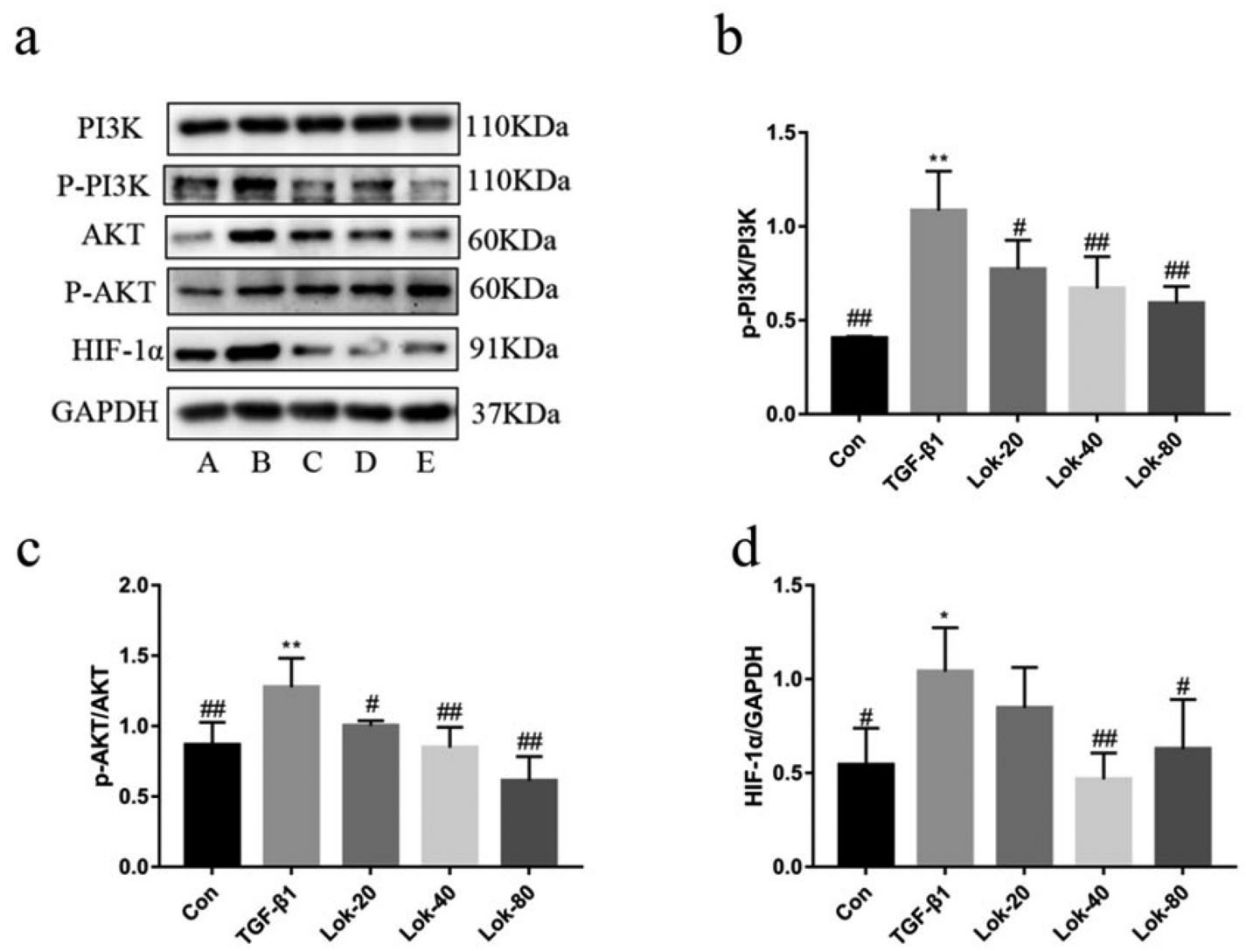
Protein levels of PI3K-AKT/HIF-1α were determined using Western blot. (a) The expression of p-PI3k, p-Akt and HIF-1α was detected by Western blot. A: control, B: TGF-β1 (10 ng/mL), C: TGF-β1 with Lok (10 ng/mL and 20 µg/mL respectively), D: TGF-β1 with Lok (10 ng/mL and 40 µg/mL, respectively), E: TGF-β1 with Lok (10 ng/mL and 80 µg/mL, respectively). Ratios of p-PI3K/PI3K (b), p-AKT/AKT (c) and HIF-1α (d) intensity. The obtained data are expressed as mean ± SD. n = 3,*p < 0.05, **p < 0.01, vs. con, ^#^*p* < 0.05, ^##^*p* < 0.01, vs. TGF-β1.

The PI3K-Akt pathway has been reported to be critical in EMT progression. Therefore, western blotting was conducted to assess whether Lok could inhibit TGF-β1-induced EMT in BEAS-2B cells through the PI3K-Akt/HIF-1α pathway. Results showed that TGF-β1 (10 ng/mL) increased p-PI3K, p-Akt, and HIF-1α expression after treatment in BEAS-2B cells for 48 h, while Lok (20, 40, 80 µg/mL) decreased p-PI3K, p-Akt, and HIF-1α expression after co-treatment with TGF-β1 in BEAS-2B cells (Figure 5). These results indicated that Lok inhibited TGF-β1-induced EMT in BEAS-2B cells through the PI3K-Akt/HIF-1α pathway.

### Lok relieved inflammation in mice with OVA-induced airway inflammation

**Figure 6.**
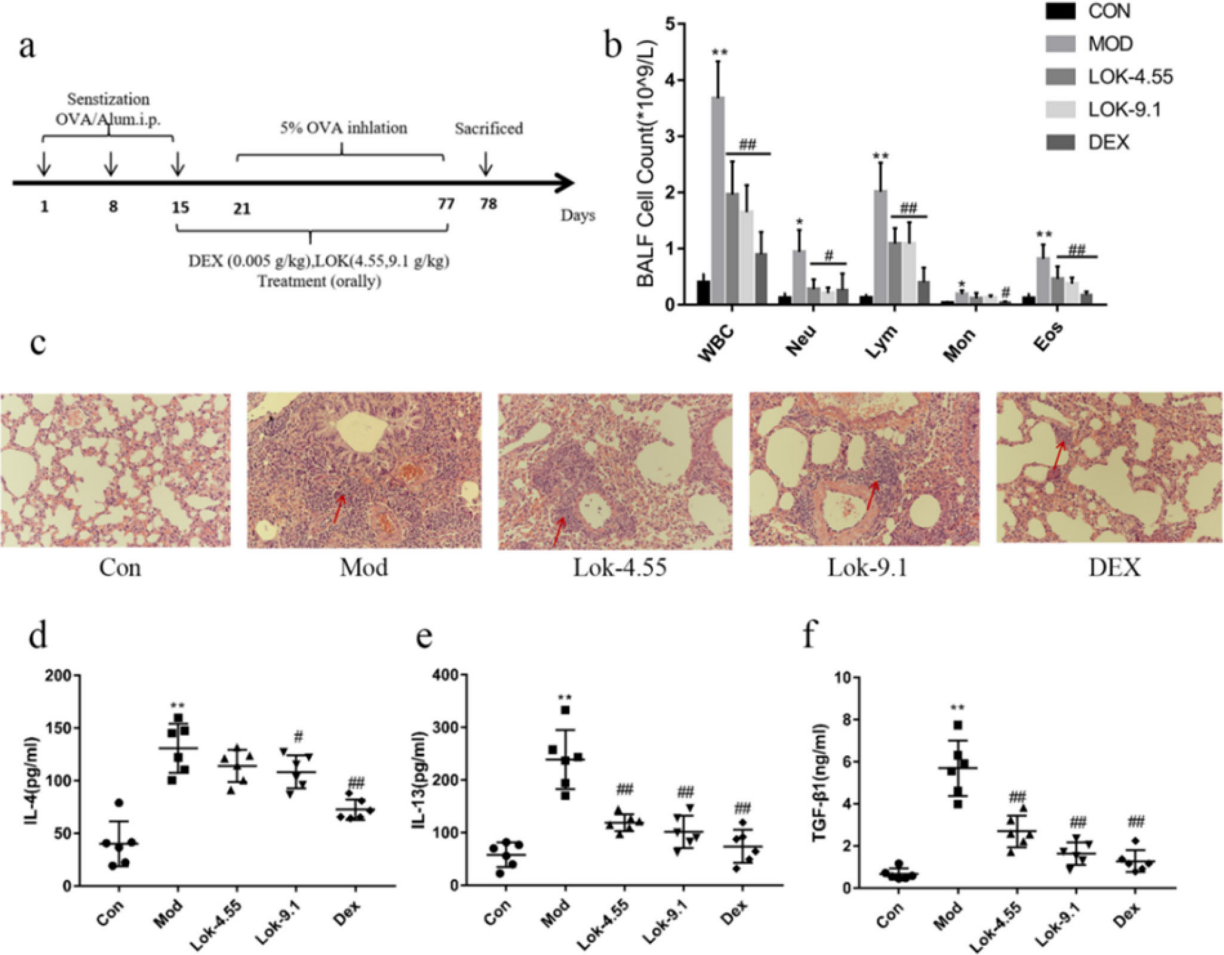
Effect of Lok on OVA-induced airway inflammation in mice. (a) Diagram representing the protocol for treating mice with Lok or Dexamethasone (Dex) following ovalbumin (OVA)-induced airway remodeling. (b) Lok decoction inhibited the inflammatory cells in BALF. Total white blood cell (WBC), lymphocyte (Lym), neutrophil (Neu), eosinophil (Eos) and monocyte (Mon) counts in BALF. (c) Histopathological examination of lung tissue was performed with hematoxylin-eosin staining (HE, magnification ×200). The expression levels of (d) IL-4 , (e) IL-13, and (f) TGF-β1 in mouse BALF were measured by ELISA. The obtained data are expressed as mean ± SD. n = 6,*p < 0.05, **p < 0.01, vs. con, ^#^*p* < 0.05, ^##^*p* < 0.01, vs. Mod.

To further explore the relationship between Lok and airway remodelling, OVA-induced asthmatic mice models were established (Figure 6(a)). Compared to the control, mice sensitized and challenged with OVA mice showed a significant increase in the total number of inflammatory cells, neutrophils (Neu), lymphocytes (Lym), monocytes (Mon) and eosinophil (Eos) in BALF (*p* < 0.01) (Figure 6(b)). Interestingly, the dexamethasone and Lok treatment group showed markedly reduced total inflammatory cells, neutrophils, lymphocytes and eosinophils in BALF compared to those treated with saline (*p* < 0.05). H&E staining of lung tissue showed that OVA sensitization and challenge induced significant inflammatory cell infiltration and bronchiolar airway thickening compared to normal controls. Importantly, treatment with dexamethasone and Lok decoction abrogated the peribronchial inflammatory changes (Figure 6(c)). The Th1/Th2 imbalance has been reported to cause airway inflammation associated with the pathogenesis of asthma. Cytokines typically associated with Th2, including interleukin (IL)-4 and IL-13, have been associated with remodelling by activating EMT transformation. We detected the pharmacological effect of Lok on Th2 immune cytokines using an ELISA kit on the BALF of the mice. We found that IL-4 and IL-13 concentrations were lower in the Lok-treated group than in the OVA group (Figure 6(d and e)). This finding indicates that Lok suppressed IL-4 and IL-13 production. Moreover, TGF-β1 levels were significantly decreased in the Lok-treated group compared to the OVA group.

### Lok relieved airway remodelling in mice with OVA-induced asthma

**Figure 7.**
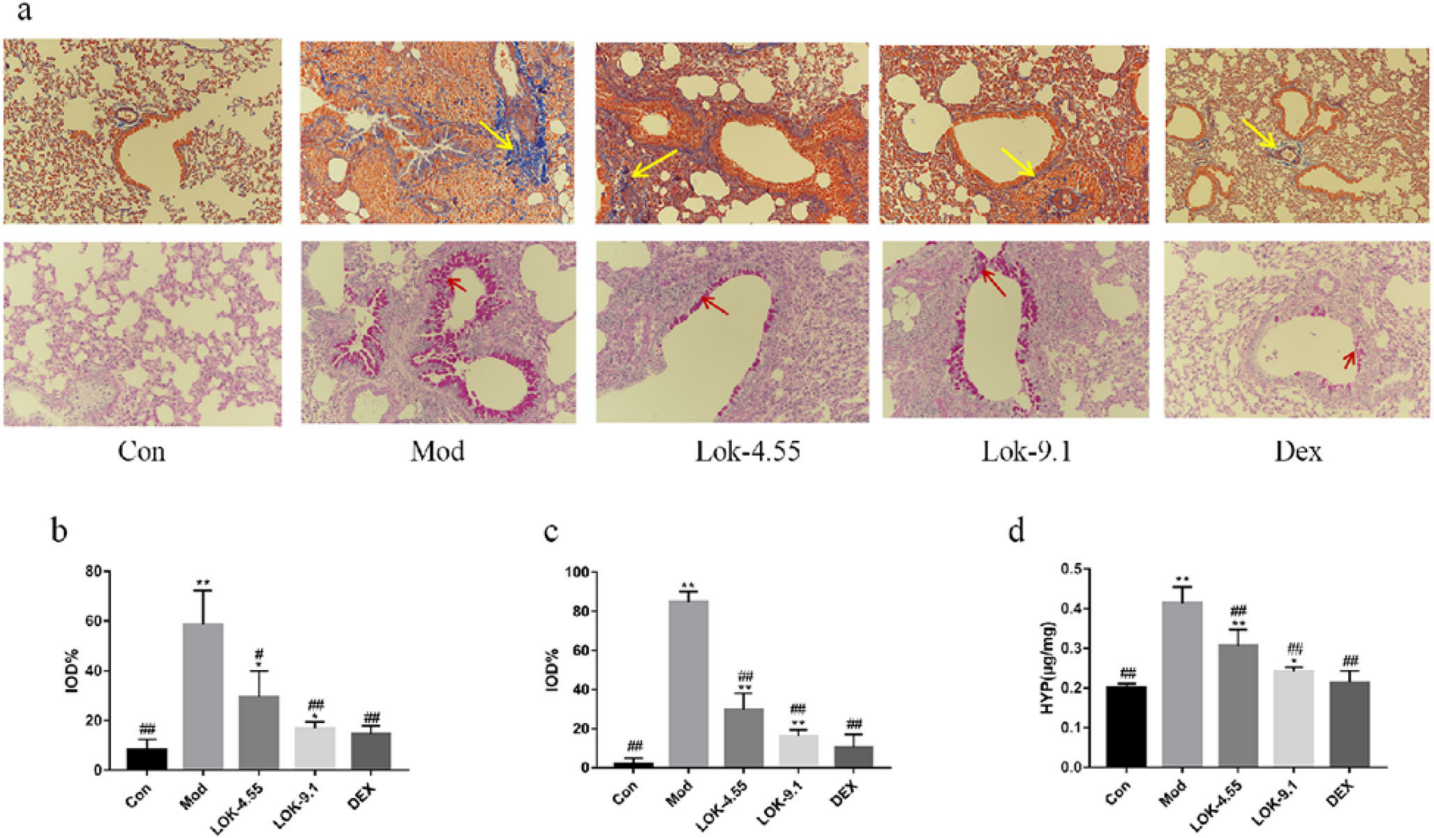
Effect of Lok treatment on OVA-induced airway remodeling in chronic asthma. (a) Masson's trichrome and Periodic Acid-Schiff (PAS) staining in lung tissue. Yellow arrows indicate collagen deposition, and red arrows indicate goblet cell proliferation in each group. Bars, 200 µm. (b) We quantified and outlined the peribronchial area stained with Masson's trichrome. (c) Percentage of PAS staining. (d) The levels of HPY expression in lung tissue. The obtained data are expressed as mean ± SD. n = 6, *p < 0.05, **p < 0.01, vs. con, ^#^*p* < 0.05, ^##^*p* < 0.01, vs. Mod.

Since airway remodelling is characterized by airway wall thickening, subepithelial collagen deposition, and excessive mucus secretion, we assessed the pathological changes in lung tissue sections. In the present study, mice exposed to OVA showed bronchiolar airway remodelling and mucus production, which resulted in airway plugging (Figure 7(a)). Next, compared to normal control mice, mice subjected to OVA showed significant changes in Masson’s trichrome stained area (*p* < 0.01) and mucus production score (*p* < 0.01) evaluated by Periodic Acid-Schiff (PAS) staining. However, DEX and Lok decoction treatment significantly reduced the Masson’s trichrome stained area compared with OVA mice (*p* < 0.05), while DEX and Lok decoction treatment significantly decreased mucus production (*p* < 0.05) (Figure 7(b and c)). Higher hydroxyproline content in lung tissue was associated with more severe pulmonary fibrosis in mice. Compared with the normal control group, the content of hydroxyproline in the lung tissue of the mod group was significantly increased compared with the control group, while the content of hydroxyproline in the lung tissues of the Lok and Dex groups was significantly decreased (Figure 7(d)).

### Lok suppressed EMT in OVA-induced asthma mice models

**Figure 8.**
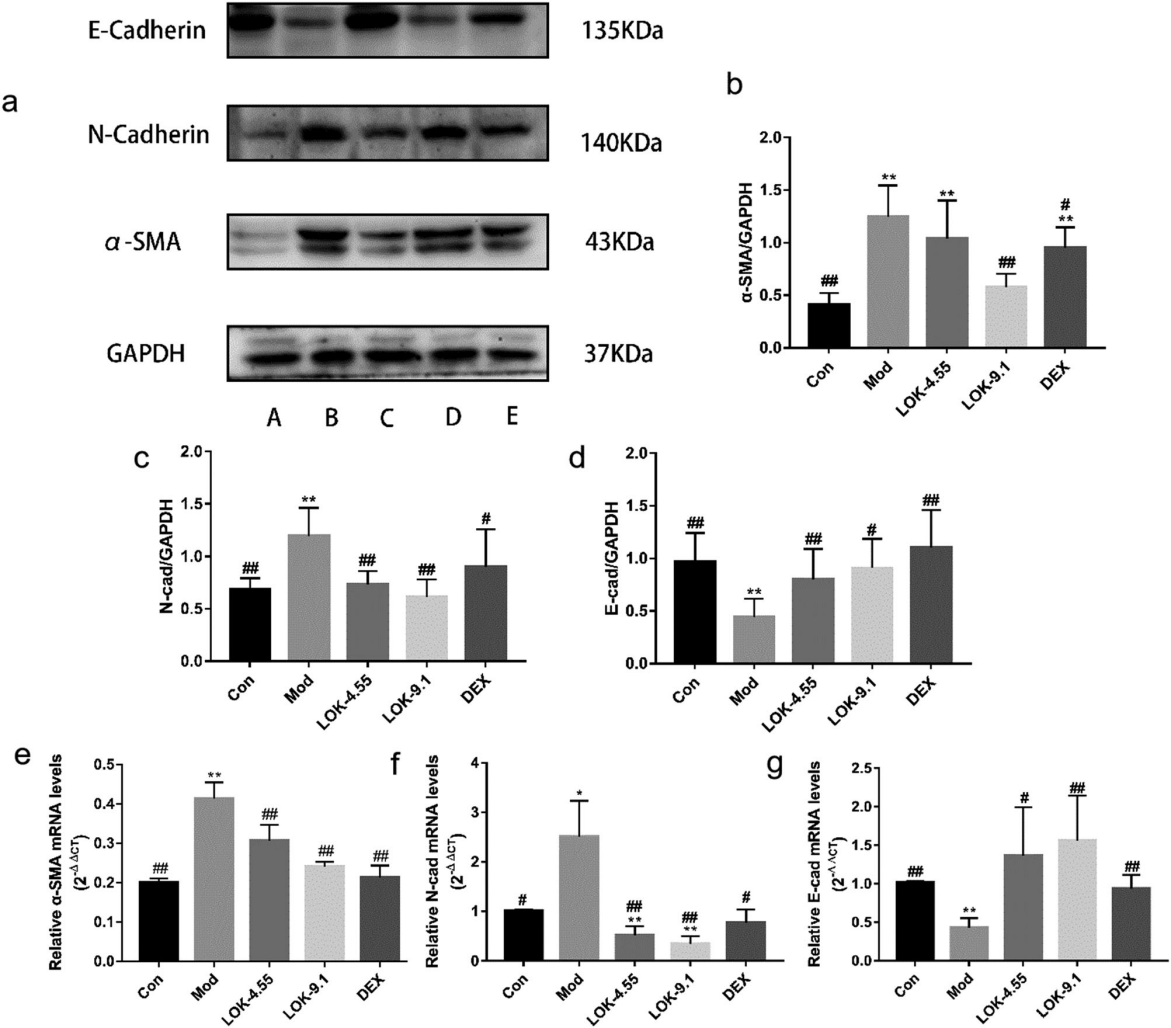
Lok decreased EMT in the lungs of mice with OVA-induced asthma. (a) The expressions of each protein were determined using Western blot. A (Con), B (Mod), C (Lok- 4.55), D (Lok- 9.1), E (Dex). Ratios of α-SMA (b), N-cadherin (c) and E-cadherin (d) intensity. The expressions of (e) α-SMA, (f) N-cadherin and (g) E-cadherin in mice lung tissues were detected by RT-qPCR. Data from six independent experiments. The obtained data are expressed as mean ± SD. n = 6, *p < 0.05, **p < 0.01, vs. con, ^#^*p* < 0.05, ^##^*p* < 0.01, vs. Mod.

It has been established that EMT plays an essential role in airway remodelling in asthma. Compared with normal mice, the lungs of chronic asthmatic mice exhibited decreased staining for the epithelial marker E-Cadherin and increased staining of mesenchymal markers, N-Cadherin and α-SMA, confirming the occurrence of EMT in the chronic asthma model. Importantly, the suppressive effect of Lok and DEX treatment on the development of EMT in chronic asthma was validated through Western blot and RT-qPCR analysis. These analyses assessed the protein levels of E-Cadherin, N-Cadherin and α-SMA in the lungs (Figure 8).

### Lok inhibits PI3K-Akt and HIF-1α signaling pathways in mice

**Figure 9.**
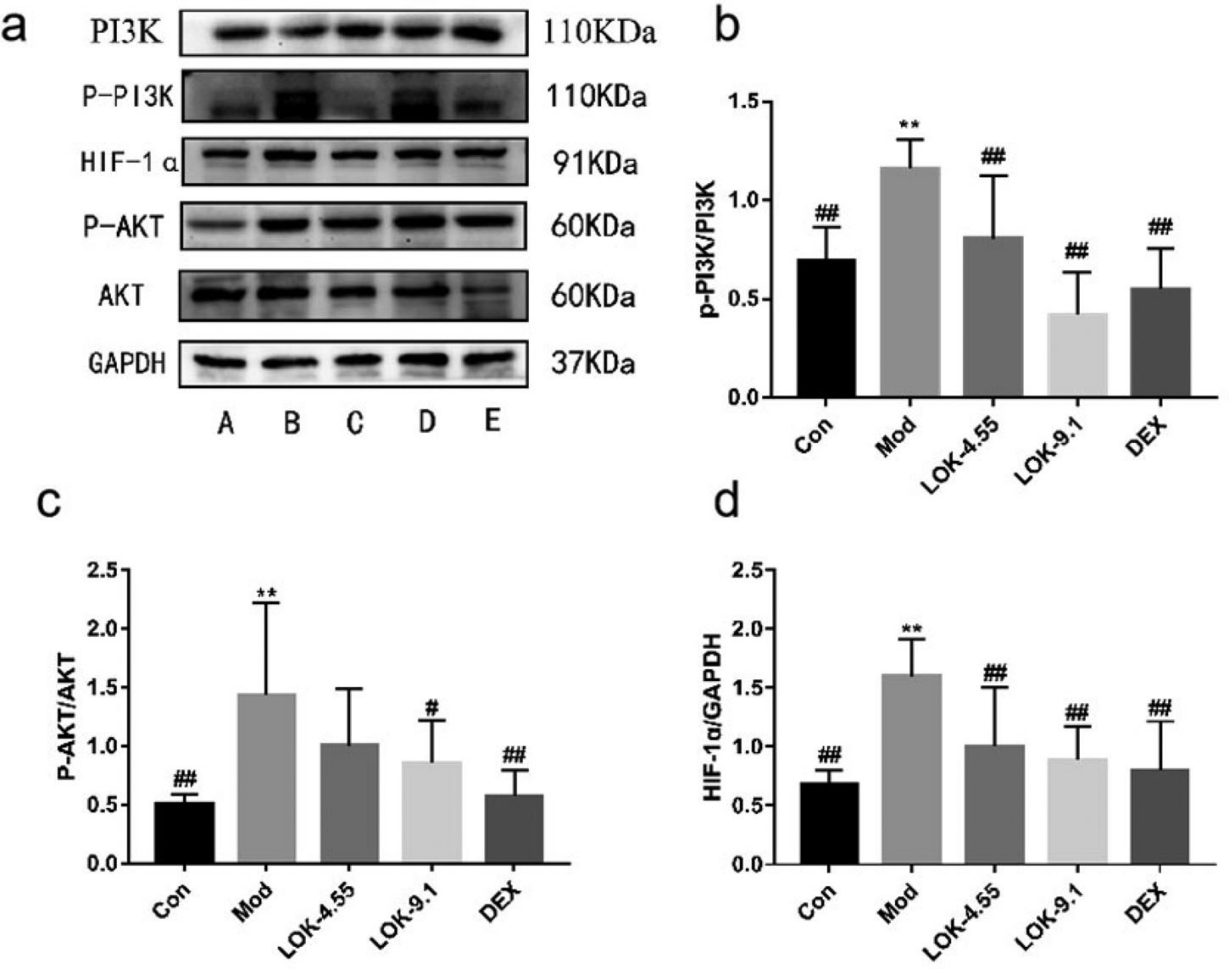
Lok reduces the expression of PI3K-AKT/HIF-1α signaling pathway-related proteins in the lungs of mice with OVA-induced asthma. (a) The expression of each protein was quantified using Western blot. A (Con), B (Mod), C (Lok-4.55), D (Lok-9.1), E (Dex). Ratios of (b) p-PI3K/PI3K, (c) p-AKT/AKT and (d) HIF-1α intensity. The obtained data are expressed as mean ± SD. n = 6, *p < 0.05, **p < 0.01, vs. con, ^#^*p* < 0.05, ^##^*p* < 0.01, vs. Mod.

Recent studies have uncovered the key role of the PI3K-Akt/HIF-1α pathway in airway remodelling in asthma. Thus, we hypothesized that Lok protects against asthma *via* modulation of the PI3K-Akt/HIF-1α pathway. Increased levels of p-PI3K, p-Akt, and HIF-1α were observed in the lungs of the asthma models, substantiating that the PI3K-Akt/HIF-1α pathway is tightly correlated with the development of asthma. As expected, Lok treatment significantly reduced p-PI3K, p-Akt and HIF-1α levels (Figure 9).

## Discussion

Airway remodelling is a structural change characterized by airway wall thickening, subepithelial collagen deposition and excessive mucus secretion (Bullone and Lavoie 2020). It has been established that airway epithelial cells are the primary targets of inhaled environmental allergens, causing the production of innate cytokines by Th2 cells that trigger allergic reactions (Sheih et al. 2017). Chronic exposure to environmental factors may lead to persistent activation of pathways involved in airway epithelial repair, such as EMT, changes in progenitor cell migration and proliferation, and abnormal redifferentiation causing airway remodelling (Ganesan and Sajjan 2013). No study has hitherto assessed the effect of Loke zupa on the changes in airway epithelial cell function induced by allergens. A previous study reported that airway remodelling might occur in the early stages of asthma and is associated with EMT dysfunction (Yao et al. 2019). Although the protective role of Lok against airway inflammation has been reported, its role in EMT in asthma remains unclear. E-cadherin is an important intercellular adhesion protein whose downregulation is a hallmark of EMT and is accompanied by the upregulation of mesenchymal markers, including fibronectin, wave protein, α-smooth muscle actin (α-SMA) and N-cadherin (Pei et al. 2019; Riemma et al. 2022; Kalluri and Weinberg 2009). In the present study, cellular experiments demonstrated that Lok treatment effectively reversed the TGF-β1-induced EMT process. In our OVA-induced asthma model, the levels of the mesenchymal marker N-Cadherin and α-SMA were increased. We observed that Lok treatment increased E-Cadherin levels and decreased N-Cadherin and α-SMA expression, indicating that EMT progression was delayed. It is highly conceivable that Lok’s antagonistic effect on airway remodelling is not only due to its anti-inflammatory properties but also to its anti-EMT properties.

Ovalbumin is one of the most abundant glycoprotein allergens responsible for Th2 immune responses in patients with asthma (Wang et al. 2014). Asthmatic airway remodelling is usually associated with chronic inflammation (Dong et al. 2021). Although the exact pathogenesis underlying airway remodelling remains unclear, it has been established that chronic inflammatory responses in the airways play an important role (Fehrenbach et al. 2017). The present study established a mouse model of OVA-induced asthma to further explore the relationship between Lok and airway remodelling. We found that airway inflammatory cell infiltration was significantly increased in the lungs of the mice model. Additionally, severe airway remodelling and changes in collagen deposition were observed. To assess the impact of remodelling inhibition on cytokines and growth factors in BALF, we examined their levels. It is widely acknowledged that chronic allergic inflammation is a driver of remodelling and pathological changes, with significant goblet cell proliferation and mucus plug formation, airway basement membrane thickening observed in the airways of mice overexpressing IL-4, IL-5 and IL-13 in the lungs (Samitas et al. 2018; Wang et al. 2020). Interestingly, IL-4 reportedly increases the expression of α-SMA, suggesting IL-4 may influence airway inflammation and collagen deposition (Bergeron et al. 2003). In contrast, it has been shown that IL-13 induces the differentiation of fibroblasts into myofibroblasts and enhances the release of type III collagen, leading to collagen deposition in the lung (Lee et al. 2001). IL-13 induces the release of TGF-β1 leading to airway fibrosis (Ingram and Kraft 2012). Our experimental results showed that Lok treatment reduced the infiltration of airway inflammatory cells in chronic asthmatic mice and decreased IL-4, IL-13 and TGF-β1 levels in BALF. In chronic asthmatic mice, mitigating these inflammatory factors reduced airway goblet cell proliferation, smooth muscle thickening, and collagen deposition.

There is an increasing consensus suggesting that EMT transformation can be regulated directly or indirectly through various molecular mechanisms, including growth factors and cytokines, such as TGF-β1, and signaling pathways, such as the PI3K signaling pathway (Ye et al. 2022; Zhou et al. 2023). PIP3 interacts with the PH structural domain of Akt, leading to the aggregation of Akt on the membrane. With the help of 3-phosphatidylinositol-dependent protein kinase1 (PDK1), Akt is activated after phosphorylation, which further phosphorylates a range of substrates, thereby affecting various cellular and physiological processes, including cell cycle, cell growth, angiogenesis, and migration, and these changes induce EMT (Ye et al. 2012). Numerous clinical trials suggest that blocking the phosphatidylinositol-3-kinase/protein kinase B signaling pathway can inhibit airway remodelling in mice through multiple mechanisms. It is widely thought that asthma activates hypoxia-responsive pathways in the body. Hypoxia-inducible factor-1α (HIF-1α) is an important factor mediating the hypoxic response, which promotes inflammation and induces activation of genes associated with airway remodelling, leading to increased inflammatory cell infiltration and fibrosis (Dahlin et al. 2018). It has been shown that dephosphorylating AKT to block the PI3K/AKT pathway reduces the expression of hypoxia-inducible factor HIF-α and vascular endothelial growth factor VEGF, thereby decreasing airway mucosal permeability, inhibiting airway edema and constriction, resulting in reduced vascular mucus secretion and decreased ovalbumin-induced airway remodelling (Zhao et al. 2018). These findings suggest that blocking the PI3K-Akt/HIF-1α pathway is a potential approach to hindering asthma airway remodelling, consistent with our results. Importantly, we found that Lok treatment significantly reduced p-PI3K, p-Akt, and HIF-1α in the lung tissue of chronically asthmatic mice. Thus, Lok may attenuate airway remodelling by inhibiting the PI3K-Akt/HIF-1α signaling pathway, thus alleviating chronic asthma airway epithelial EMT transformation.

Our findings suggest that Lok significantly prevents EMT progression by reducing TGF-β1 production in epithelial cells, alleviating airway inflammation and remodelling in experimental asthma models by suppressing EMT *via* downregulating PI3K-Akt and HIF-1α expression. However, the limitations of our study should be acknowledged. Since the direct target protein of Lok in the body has not been identified, we evaluated the effects of Lok on EMT through histopathology and cell function experiments. Indeed, more comprehensive basic and clinical studies are needed to evaluate the efficacy and mechanism of airway remodelling. Besides, we did not measure a known inhibitor of the PI3K-AKT and HIF-1α signaling pathways for comparison. It should be borne in mind that other related pathways may affect asthma pathophysiology and were not studied. These pathways should be acknowledged and addressed in future research studies.

## Conclusions

In brief, this study yielded several conclusions. Firstly, Lok displayed a protective role in an *in vitro* BEAS-2B model and asthma mice, exhibiting notable anti-inflammatory effects and improvements in EMT. Besides, Lok may attenuate airway remodelling by inhibiting the PI3K-Akt/HIF-1α signalling pathway, thus alleviating chronic asthma airway epithelial EMT transformation. Our future studies will focus on the relevant mechanisms of Lok in asthma to better understand its therapeutic effect.

## References

[CIT0001] Abduwaki M, Abdukadir A, Eziz A, Nasir G, Hojahmat H, Nurahmat M. 2017. Uygur medicine to treat asthma preparations LuoouKe zupa research overview. XinJiang Med J. 47:123–128. Chinese.

[CIT0002] Aihaiti K, Li J, Yaermaimaiti S, Liu L, Xin X, Aisa HA. 2022. Non-volatile compounds of *Hyssopus cuspidatus* Boriss. and their antioxidant and antimicrobial activities. Food Chem. 374:131638. doi: 10.1016/j.foodchem.2021.131638. 34839965

[CIT0003] Bullone M, Lavoie JP. 2020. The equine asthma model of airway remodelling: from a veterinary to a human perspective. Cell Tissue Res. 380(2):223–236. doi: 10.1007/s00441-019-03117-4. 31713728

[CIT0004] Bergeron C, Pagé N, Joubert P, Barbeau B, Hamid Q, Chakir J. 2003. Regulation of procollagen I (alpha1) by interleukin-4 in human bronchial fibroblasts: a possible role in airway remodelling in asthma. Clin Exp Allergy. 33(10):1389–1397. doi: 10.1046/j.1365-2222.2003.01785.x. 14519145

[CIT0005] Chung KF. 2017. Clinical management of severe therapy-resistant asthma. Expert Rev Respir Med. 11(5):395–402. doi: 10.1080/17476348.2017.1317597. 28388228

[CIT0006] Caramori G, Nucera F, Mumby S, Bello FL, Adcock IM. 2022. Corticosteroid resistance in asthma: cellular and molecular mechanisms. Mol Aspects Med. 85:100969. doi: 10.1016/j.mam.2021.100969. 34090658

[CIT0007] Derendorf H, Nave R, Drollmann A, Cerasoli F, Wurst W. 2006. Relevance of pharmacokinetics and pharmacodynamics of inhaled corticosteroids to asthma. Eur Respir J. 28(5):1042–1050. doi: 10.1183/09031936.00074905. 17074919

[CIT0008] Dahlin A, Qiu W, Litonjua AA, John JL, Mayumi T, Michiaki K, Irvin CG, Peters SP, Wu AC, Weiss ST, et al. 2018. The phosphatidylinositide3-kinase (PI3K) signaling pathway is a determinant of zileuton response in adults with asthma. Pharmacogenomics J. 18(5):665–677. doi: 10.1038/s41397-017-0006-0. 29298996 PMC6150906

[CIT0009] Dong LY, Wang Y, Zheng TT, Pu YN, Ma YB, Qi X, Zhang WZ, Xue F, Shan ZR, Liu JM, et al. 2021. Hypoxic hUCMSC-derived extracellular vesicles attenuate allergic airway inflammation and airway remodelling in chronic asthma mice. Stem Cell Res Ther. 12(1):4. doi: 10.1186/s13287-020-02072-0. 33407872 PMC7789736

[CIT0010] Fehrenbach H, Wagner C, Wegmann M. 2017. Airway remodelling in asthma: what really matters. Cell Tissue Res. 367(3):551–569. doi: 10.1007/s00441-016-2566-8. 28190087 PMC5320023

[CIT0011] Gosens R, Grainge C. 2015. Bronchoconstriction and airway biology: potential impact and therapeutic opportunities. Chest. 147(3):798–803. doi: 10.1378/chest.14-1142. 25732446

[CIT0012] Grainge C, Lau LK, Ward JA, Dulay V, Lahiff G, Wilson S, Holgate S, Davies DE, Howarth PH. 2011. Effect of bronchocon-striction on airway remodelling in asthma. N Engl J Med. 364(21):2006–2015. doi: 10.1056/NEJMoa1014350. 21612469

[CIT0013] Ganesan S, Sajjan US. 2013. Repair and remodelling of airway epithelium after injury in chronic obstructive pulmonary disease. Curr Respir Care Rep. 2:145–154. doi: 10.1007/s13665-013-0052-2. PMC3811962 24187653

[CIT0014] Ingram JL, Kraft M. 2012. IL-13 in asthma and allergic disease: asthma phenotypes and targeted therapies. J Allergy Clin Immunol. 130(4):829–842. doi: 10.1016/j.jaci.2012.06.034. 22951057

[CIT0015] Kalluri RH, Weinberg RA. 2009. The basics of epithelial-mesenchymal transition. J Clin Invest. 119(6):1420–1428. doi: 10.1172/JCI39104. 19487818 PMC2689101

[CIT0016] Lee CG, Homer RJ, Zhu Z, Lanone S, Wang X, Koteliansky V, Shipley JM, Gotwals P, Noble P, Chen Q, et al. 2001. Interleukin-13 induces tissue fibrosis by selectively stimulating and activating transforming growth factor beta (1). J Exp Med. 194(6):809–821. doi: 10.1084/jem.194.6.809. 11560996 PMC2195954

[CIT0017] Li W, Li L, Wang XM, Diao JJ, Wang XL. 2021. Determination of total polyphenols and total flavonoids in compound Lok and study on antioxidant activity. J Xinjiang Med Univ. 44:842–845. Chinese.

[CIT0018] Mohammadtursun N, Li QP, Abuduwaki M, Jiang S, Zhang H, Sun J, Dong JC. 2020. Loki zupa alleviates inflammatory and fibrotic responses in cigarette smoke induced rat model of chronic obstructive pulmonary disease. Chin Med. 15:92. doi: 10.1186/s13020-020-00373-3. 32874197 PMC7457355

[CIT0019] Pei DQ, Shu XD, Diagne AG, Thiery JP. 2019. Mesenchymal-epithelial transition in development and reprogramming. Nat Cell Biol. 21(1):44–53. doi: 10.1038/s41556-018-0195-z. 30602762

[CIT0020] Riemma MA, Cerqua I, Romano B, Irollo E, Bertolino A, Camerlingo R, Granato E, Rea G, Scala S, Terlizzi M, et al. 2022. Sphingosine-1-phosphate/TGF-β axis drives epithelial mesenchymal transition in asthma-like disease. Br J Pharmacol. 179(8):1753–1768. doi: 10.1111/bph.15754. 34825370 PMC9306821

[CIT0021] Samitas K, Carter A, Kariyawasam HH, Xanthou G. 2018. Upper and lower airway remodelling mechanisms in asthma, allergic rhinitis and chronic rhinosinusitis: the one airway concept revisited. Allergy. 73(5):993–1002. doi: 10.1111/all.13373. 29197105

[CIT0022] Sheih A, Parks WC, Ziegler SF. 2017. GM-CSF produced by the airway epithelium is required for sensitization to cockroach allergen. Mucosal Immunol. 10(3):705–715. doi: 10.1038/mi.2016.90. 27731325 PMC5389932

[CIT0023] Wang HS, Kim JY, Park H, Jeong J, Hyun H, Yoon TJ, Park H-Y, Choi H-D, Kim HH. 2014. Cleavage of the terminal *N*-acetylglucosamine of egg-white ovalbumin *N*-glycans significantly reduces IgE production and Th2 cytokine secretion. Biochem Biophys Res Commun. 450(4):1247–1254.,. doi: 10.1016/j.bbrc.2014.06.101. 25010643

[CIT0024] Wang XZ, Xu CY, Ji JY, Cai YQ, Shu YY, Chao YQ, Wu XL, Zou CH, Wu XM, Tang LF. 2020. IL-4/IL-13 upregulates Sonic hedgehog expression to induce allergic airway epithelial remodelling. Am J Physiol Lung Cell Mol Physiol. 318(5):L888–L899. doi: 10.1152/ajplung.00186.2019. 32130032

[CIT0025] Wei Y, Abduwaki M, Li MH, Luo QL, Sun J, Lv YB, Nurahmat M, Dong JC. 2016. Lok (Luooukezupa) decoction reduced airway inflammation in an OVA-induced asthma mouse model. Chin Med. 11:22. doi: 10.1186/s13020-016-0094-9. 27134644 PMC4851804

[CIT0026] Wei L, Gou XL, Su B, Han HQ, Guo TT, Liu L, Wang L, Zhang L, Chen WB, Lina W, et al. 2022. Mahuang decoction attenuates airway inflammation and remodelling in asthma via suppression of the SP1/FGFR3/PI3K/AKT axis. Drug Des Devel Ther. 16:2833–2850. doi: 10.2147/DDDT.S351264. PMC9427210 36051156

[CIT0027] Wuniqiemu T, Qin J, Teng F, Nabijan M, Cui J, Yi L, Tang W, Zhu XY, Abduwaki M, Nurahmat M, et al. 2021. Quantitative proteomic profiling of targeted proteins associated with Lok decoction treatment in ova-induced asthmatic mice. J Ethnopharmacol. 266:113343. doi: 10.1016/j.jep.2020.113343. 32991972

[CIT0028] Xie C, Gul A, Yu H, Huang X, Deng LL, Pan Y, Ni SS, Nurahmat M, Abduwaki M, Luo QL, et al. 2022. Integrated systems pharmacology and transcriptomics to dissect the mechanisms of Loki Zupa decoction in the treatment of murine allergic asthma. J Ethnopharmacol. 294:115351. doi: 10.1016/j.jep.2022.115351. 35533913

[CIT0029] Ye B, Jiang LL, Xu HT, Zhou DW, Li ZS. 2012. Expression of PI3K/Akt pathway in gastric cancer and its blockade suppresses tumor growth and metastasis. Int J Immunopathol Pharmacol. 25(3):627–636. doi: 10.1177/039463201202500309. 23058013

[CIT0030] Ye ZX, Jiang YF, Wu JL. 2022. DNMT3B attenuated the inhibition of TET3 on epithelial-mesenchymal transition in TGF-β1-induced ovarian cancer by methylating the TET3 promoter. Reprod Biol. 22(4):100701. doi: 10.1016/j.repbio.2022.100701. 36242939

[CIT0031] Yuan FJ, Liu R, Hu M, Rong XJ, Bai LP, Xu L, Mao Y, Hasimu H, Sun YH, He JH. 2019. JAX2, an ethanol extract of *Hyssopus cuspidatus* Boriss., can prevent bronchial asthma by inhibiting MAPK/NF-kappaB inflammatory signaling. Phytomedicine. 57:305–314. doi: 10.1016/j.phymed.2018.12.043. 30807985

[CIT0032] Yao L, Wang S, Wei P, Bao K, Yuan W, Wang X, Zheng J, Hong M. 2019. Huangqi-Fangfeng protects against allergic airway remodelling through inhibiting epithelial-mesenchymal transition process in mice via regulating epithelial derived TGF-b1. Phytomedicine. 64:153076. doi: 10.1016/j.phymed.2019.153076. 31473579

[CIT0033] Zhao X, Yu FQ, Huang XJ, Xu BY, Li YL, Zhao XY, et al. 2018. Azithromycin influences airway remodelling in asthma via the PI3K/Akt/MTOR/HIF-1α/VEGF pathway. J Biol Regul Homeost Agents. 32:1079–1088. 30334401

[CIT0034] Zhou B, Zhou X, Zhan C, Jin M, Yan S. 2023. FAM83A promotes the progression and metastasis of human pancreatic neuroendocrine tumors by inducing the epithelial-mesenchymal transition via the PI3K/AKT and ERK pathways. J Endocrinol Invest. 46(6):1115–1130. doi: 10.1007/s40618-022-01959-4. 36344884

